# Granulomatosis With Polyangiitis Presenting As Orbital Apex Syndrome

**DOI:** 10.7759/cureus.64087

**Published:** 2024-07-08

**Authors:** Hannah Ulatowski, Andrea Bequest, Alisha Sharma, Pinky Jha

**Affiliations:** 1 Internal Medicine, Medical College of Wisconsin, Milwaukee, USA

**Keywords:** cocaine levamisole-induced vasculitis, sino-nasal mass, c-anca vasculitis, orbital apex syndrome, granulomatosis with polyangiitis (gpa)

## Abstract

Granulomatosis with polyangiitis (GPA) is a rare small-vessel vasculitis that typically presents with a triad of sinonasal, pulmonary, and renal symptoms. Here, we present the case of a 43-year-old female with a history of substance use disorder who presented with vision changes and worsening left eye pain over five days. Previous evaluations raised concerns about GPA versus cocaine-induced vasculitis, but diagnostic confirmation was hindered by a lack of medical follow-up. Prompt multidisciplinary intervention led to significant improvement following steroid therapy and IV antibiotics, and the patient was ultimately diagnosed with a high GPA. This case highlights the complexities involved in diagnosing and managing GPA presenting as orbital apex syndrome, particularly in patients with comorbidities and non-adherence to medical follow-up.

## Introduction

Granulomatosis with polyangiitis (GPA, formerly Wegener’s granulomatosis) is a small-vessel vasculitis that belongs to the antineutrophil cytoplasmic antibody (ANCA)-associated vasculitides. GPA is a relatively rare disease, affecting an estimated 10-20 people per million, with a predilection for individuals aged 40-60 [1]. It is typically characterized by a triad of sinonasal, pulmonary, and renal symptoms, most commonly presenting with chronic sinusitis or rhinitis. The diagnosis is confirmed by a biopsy of affected tissues showing necrotizing granulomatous inflammation [1].

The clinical presentation of GPA can be variable but frequently includes nonspecific symptoms such as arthralgias, fever, cough, and cutaneous manifestations. Ophthalmic involvement is a well-recognized manifestation, reported in up to 58% of cases [2]. This can manifest as either local inflammation of ocular structures or the contiguous spread of inflammatory processes from adjacent paranasal sinuses or the nasopharynx, leading to orbital inflammation.

Orbital apex syndrome (OAS) is a rare manifestation characterized by dysfunction of the optic nerve, the third, fourth, and sixth cranial nerves, and the ophthalmic division of the fifth cranial nerve. OAS can result from various etiologies, including inflammation, infection, neoplasm, vascular disease, or trauma [3]. This case report describes a rare presentation of GPA as OAS.

## Case presentation

A 43-year-old female presented to the emergency department with chief complaints of vision changes, worsening left eye pain, and vision loss over the preceding five days. She described the sensation as if a screen had been placed over her left eye. She also reported recurrent fevers and chills. The patient had a significant medical history, including hypertension, chronic anemia, substance use disorder, and nasal septal perforation. Previous evaluations had raised concerns about GPA versus cocaine-induced vasculitis. During the workup for these, she was found to have positive C-ANCA testing. She had also previously experienced binocular diplopia over the preceding two years, for which she saw an ophthalmologist. A nasal cavity mass was found, raising concerns about bacterial sinusitis versus GPA. Despite recommendations from multiple physicians, she did not follow up with rheumatology.

A few days prior to this admission, she presented at an alternate emergency department for the same concern of visual deterioration. Subsequent evaluation revealed visual acuity of 20/250 OS with an intraocular pressure of around 30 mmHg. She was administered timolol and dorzolamide and discharged home. However, her symptoms continued to worsen, which prompted her to return to the emergency room.

**Figure 1:**
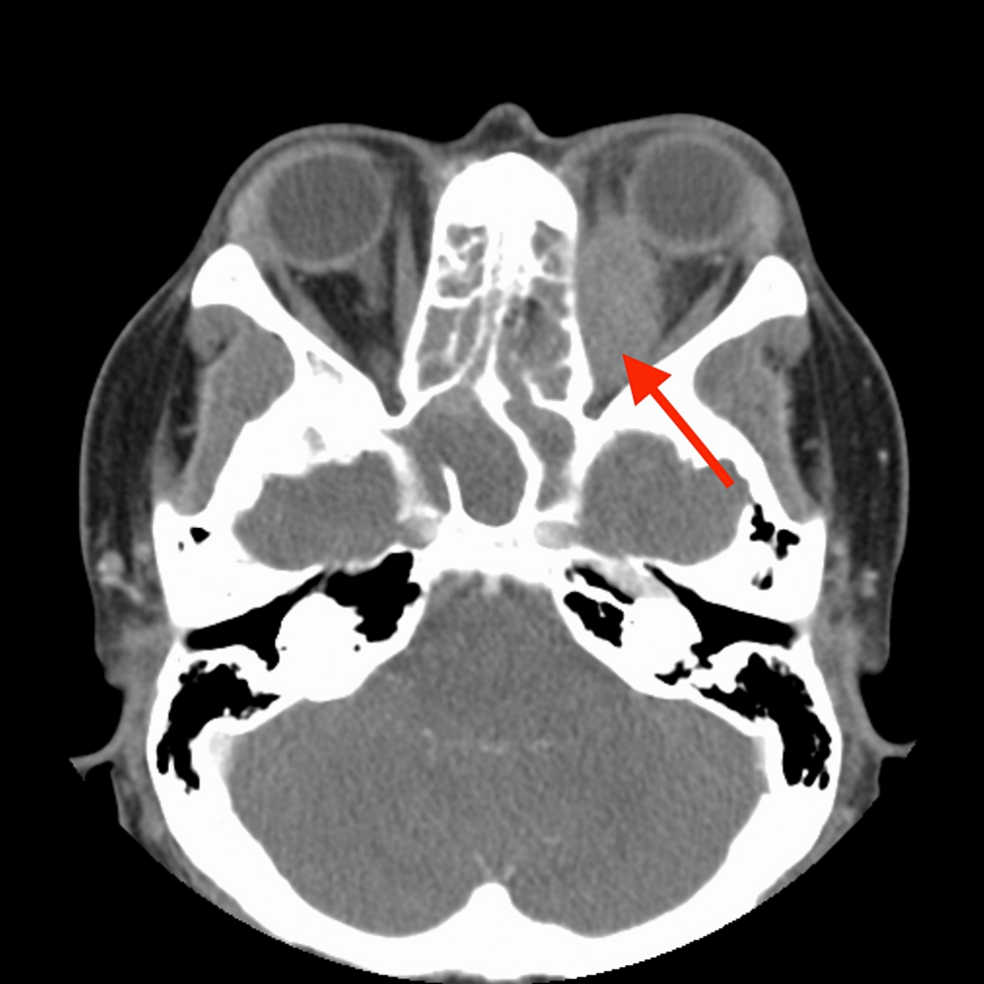
CT facial bones with contrast axial CT: computed tomography

**Figure 2:**
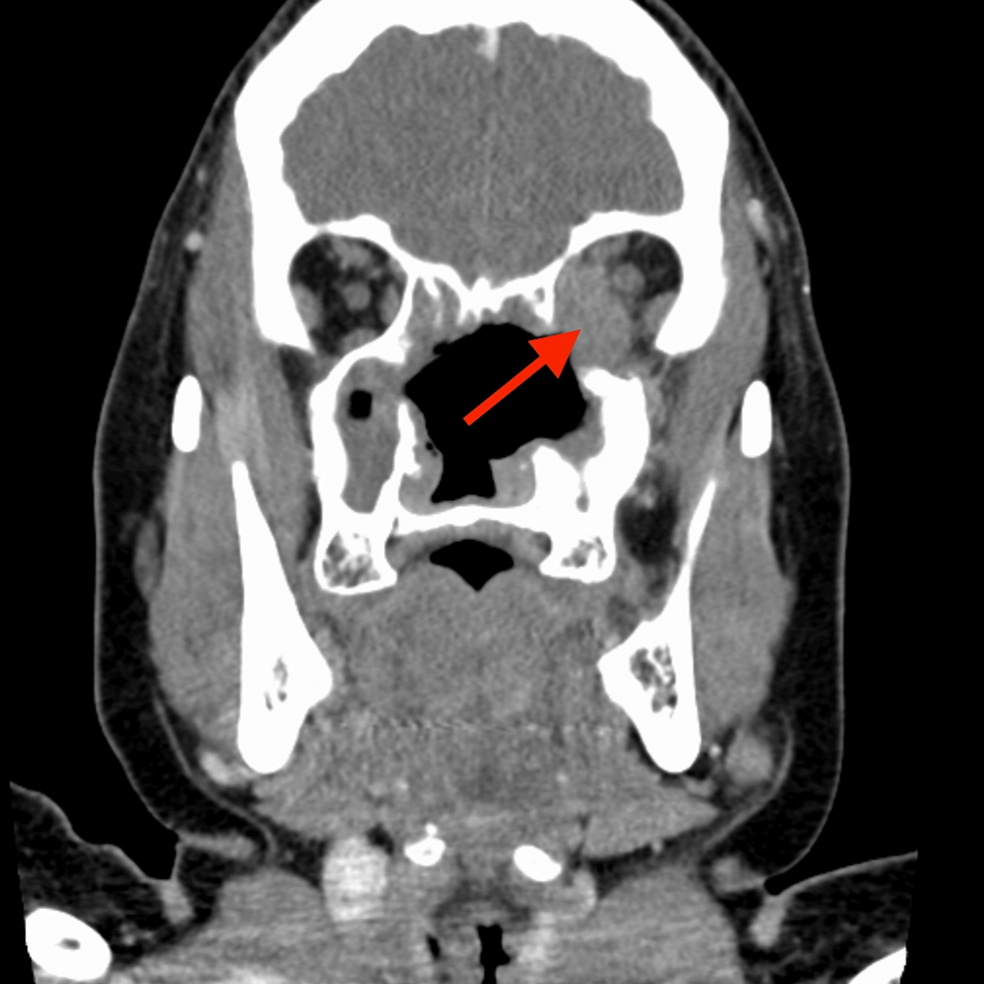
CT facial bones with contrast coronal CT: computed tomography

At presentation, the patient was afebrile with an elevated blood pressure of 181/100 mmHg. Initial laboratory investigations revealed elevated white blood cell count (15.0 cells per liter), erythrocyte sedimentation rate (89 mm/hr), and C-reactive protein (4.78 mg/dL). Urinalysis was unremarkable. Imaging studies, including CT of the facial bones (Figures 1-2) and MRI, demonstrated osseous defects involving the left orbit and facial sinuses, with thickened soft tissue extending into the left orbit and orbital canal. MRI revealed a large left orbital mass (43 mm in diameter) associated with edema in the posterior optic nerve and surrounding soft tissue.

In the emergency department, the patient received symptomatic management, including clonidine, Dilaudid, and dorzolamide/timolol eye drops, alongside IV cefepime and vancomycin for broad-spectrum coverage. Ophthalmology was urgently consulted. Examination at this presentation revealed complete ophthalmoplegia and decreased vision (20/250) in the left eye. Slit lamp examination demonstrated a large congested, dilated, and tortuous corkscrew vessel nasally, along with diffuse punctuate epithelial erosions and a stromal infiltrate with an overlying epithelial defect measuring 0.5 mm. Tables 1-3 highlight additional findings from eye exams. Overall, this presentation was highly suggestive of OAS with compressive optic neuropathy of the left eye.

**Table 1:** Ophthalmic examination at admission OD: oculus dexter (right eye), OS: oculus sinister (left eye)

	OD	OS
Pupils	3 mm in dark, 2 mm in light, no relative afferent pupillary defect	3 mm in dark, 2 mm in light, 1+ relative afferent pupillary defect
Intraocular pressure (after timolol and dorzolamide)	16 mmHg	17 mmHg
Motility and alignment	Full	-4 supraduction, -4 infraduction, -4 adduction, -4 abduction
Color vision (Ishihara plates)	11/11	0/11

**Table 2:** Slit lamp exam at admission OD: oculus dexter (right eye), OS: oculus sinister (left eye), WNL: within normal limits, NVI: neovascularization of the iris

Slit lamp exam	OD	OS
Lids/lashes	WNL	Hyperpigmentation of left upper periorbita
Conjunctiva/sclera	Trace injection	Trace injection, large congested dilated and tortuous corkscrew vessel nasally
Cornea	2+ diffuse punctuate epithelial erosions	2+ diffuse punctuate epithelial erosions, 0.5 mm round stromal infiltrate at 11:00 with a small overlying epithelial defect, no other infiltrates, negative Seidel
Anterior chamber	Deep (grade IV by Van Herick), quiet	Deep (grade IV by Van Herick), quiet
Iris	Round, regular, no NVI, persistent pupillary membrane	Round, regular, no NVI
Lens	Clear	Clear
Anterior vitreous	Clear	Clear

**Table 3:** Dilated fundus exam at admission OD: oculus dexter (right eye), OS: oculus sinister (left eye)

Dilated fundus exam	OD	OS - limited views
Cup to disc ratio	0.4	0.4
Optic nerve head	Pink, sharp margins, no swelling or pallor, healthy rim	Pink, sharp margins, no swelling, trace temporal pallor
Vessels	Normal	Normal
Macula	Flat, no heme/lipid/fluid	Flat, no heme/lipid/fluid
Periphery	Attached 360	Attached 360

Given the severity of the clinical presentation, the patient was admitted for further evaluation and management. Rheumatology was consulted and a CT of the chest was recommended, which revealed a stable subpleural left lower lobe with a 6 mm nodular density consistent with prior imaging. Quantiferon, hepatitis B, and hepatitis C labs were collected to rule out infectious etiologies, which all came back negative. Autoimmune workup was done with C-ANCA, p-ANCA, MPO, and PR3 levels. Prior to these results, she was started on IV methylprednisolone at 1000 mg daily for three days, which was eventually transitioned to oral prednisone at 80 mg/day.

On her last day of admission, there was a significant improvement in visual acuity following steroids and IV ampicillin/sulbactam. She also began to regain extraocular motility with adduction and infraduction from her complete ophthalmoplegia at admission. The responsiveness to steroids, in addition to her previously positive C-ANCA test, supported the diagnosis of GPA.

Upon stabilization of vision, the patient was discharged home with a follow-up appointment with rheumatology. Steroids were continued at discharge with a plan to taper over the next two months. Labs that resulted following discharge included C-ANCA titer, which was 1:320, serine protease 3 Ab IgG, which was 166, and MPO, which was negative. Positive C-ANCA testing, inflammation of the nasal/paranasal sinuses, and a stable pulmonary nodule on imaging met the criteria for the diagnosis of GPA [4]. The patient was given instructions to follow up with rheumatology, where they would discuss adding rituximab to the patient’s treatment.

## Discussion

OAS is a rare neurological condition that presents with painful external ophthalmoplegia with vision loss [5]. Diagnosing OAS, identifying the underlying cause, and treating OAS require multidisciplinary collaboration. Early recognition is key to preventing further complications. Once an underlying cause is identified, OAS typically responds to appropriate treatment within 72 hours, although the prognosis greatly depends on the cause [5].

The diagnosis of GPA can also be challenging and requires prompt recognition to prevent permanent organ damage. In a study by Jiang et al., all cases of GPA presenting with optic involvement were misdiagnosed for 2-36 months prior to a definitive diagnosis [6]. Clinicians should have a high suspicion for GPA in patients with both ocular and sinus inflammation/involvement. The presentation of OAS without a previous diagnosis of GPA, concurrent substance use disorder, and poor medical follow-up all complicated the diagnosis in our patient.

This patient had a known history of cocaine use, with symptoms such as nasal perforation that aligned with this diagnosis. Cocaine-induced vasculitis shares numerous clinical features with GPA, including systemic inflammation as well as nasal and ocular symptoms often secondary to direct vasoconstriction [7]. In addition, approximately 60% of cocaine in the United States is thought to contain levamisole, an anti-helminthic that has independently been shown to cause vasculitis [8].

Cocaine use has also been shown to induce ANCA production [9]. Although it was previously thought that dual positivity for MPO and PR3-ANCA is characteristic of cocaine/levamisole-induced vasculitis, a recent case series analysis suggested this might not necessarily be pathognomonic [8]. This case series highlighted alternative presentations, including 11 out of 42 cases positive for C-ANCA. Additionally, nasal biopsies did not show the characteristic granulomata of GPA, and renal/pulmonary involvement is exceedingly rare in cocaine/levamisole-induced vasculitis compared to GPA [8]. Positive C-ANCA testing, response to steroids, and lung involvement in our patient all supported a diagnosis of GPA.

Furthermore, the presentation of OAS raised concerns for alternative diagnoses, particularly infectious and neoplastic etiologies [10]. Given the patient’s history of self-reported fevers and chills, infectious causes were high on the differential. Testing came back negative, however, and treatment with steroids proving effective supported an inflammatory cause. This complex clinical presentation required that ophthalmology work closely with rheumatology and the hospital medicine team to address this patient’s OAS.

Finally, despite previous recommendations for rheumatologic evaluation, the patient's non-adherence delayed the diagnosis and initiation of appropriate treatment. There were multiple points where physicians were concerned about GPA, and prompt evaluation might have prevented this presentation of vision loss. This highlights the importance of patient education and engagement in their healthcare, particularly in chronic and potentially debilitating conditions like GPA.

Treatment of GPA is often combined therapy with corticosteroids, cyclophosphamide, or rituximab [1]. However, relapses are common. Fortunately, the patient showed significant improvement in visual acuity and extraocular motility within 48 hours of initiating steroid therapy and IV antibiotics. This underscores the importance of early intervention in GPA-associated OAS to prevent permanent vision loss and cranial nerve dysfunction. A previous case report by Shunmugam et al. highlighted the severity of GPA presenting with OAS. In this case, vision changes were resistant to treatment and eventually resulted in permanent vision loss [11]. It is thought that visual prognosis is related to either the severity of GPA or the length of time between diagnosis and initiation of treatment [12]. Long-term management and monitoring are essential to reduce relapses and optimize outcomes, especially in patients with underlying substance use disorders and poor medical adherence.

## Conclusions

This case highlights the complexities involved in diagnosing GPA presenting as OAS, particularly in patients with comorbidities and non-adherence to medical follow-up. The case also adds to the body of literature on atypical presentations of GPA. Multidisciplinary collaboration and patient engagement are crucial for timely diagnosis, the initiation of appropriate treatment, and the prevention of irreversible complications.
